# Supplementary figures and images for: Signatures in SARS-CoV-2 spike protein conferring escape to neutralizing antibodies
Source: PLoS Pathog. 2021 Aug 5;17(8):e1009772. doi: 10.1371/journal.ppat.1009772 (PMC8341613; doi:10.1371/journal.ppat.1009772)

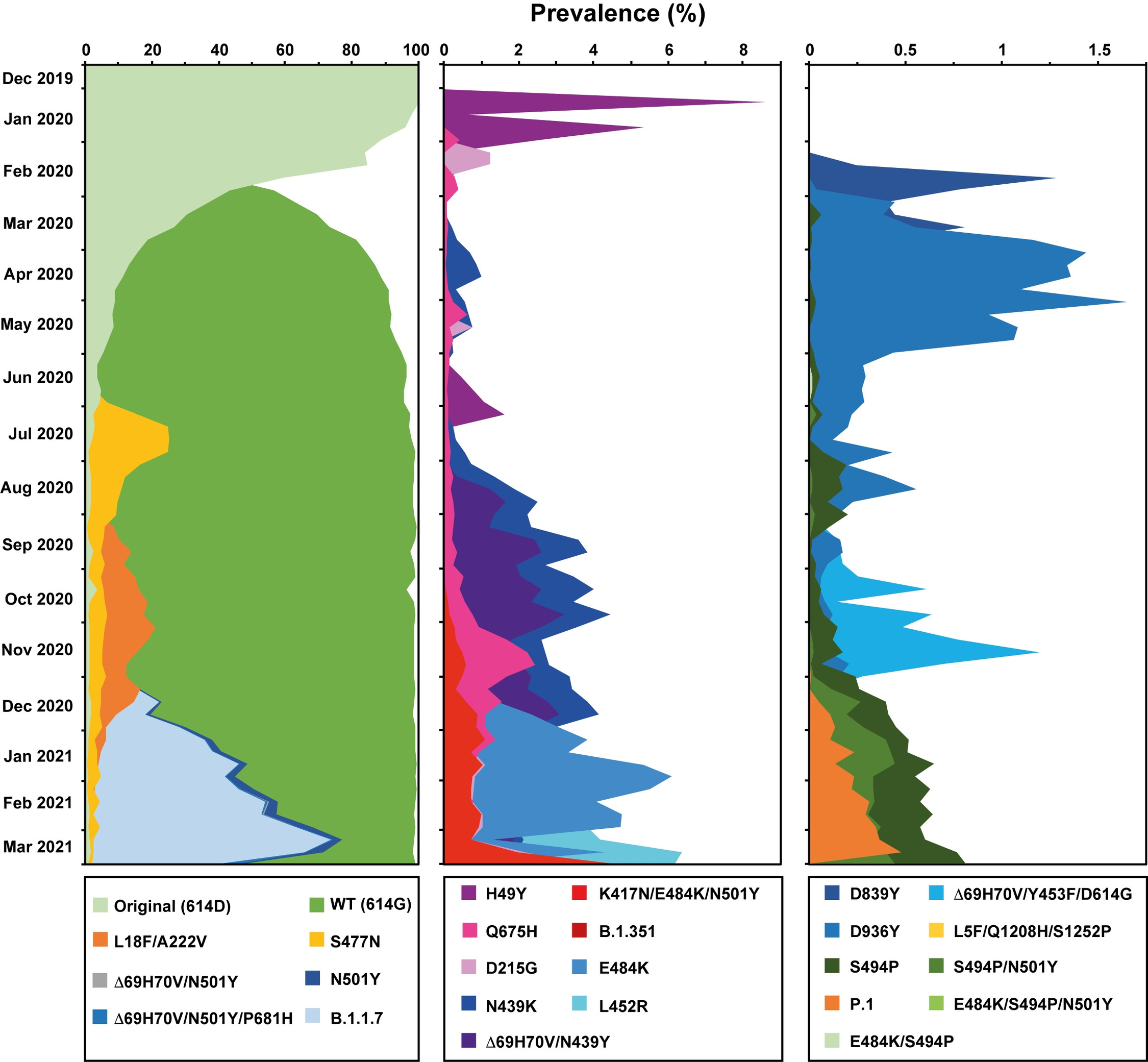

Supplement: S1 Fig — To investigate the global frequency of SARS-CoV-2, the variant surveillance dataset was retrieved on March 25th, 2021, from GISAID (Shu Y, McCauley J., 2017). From this dataset and using text mining tools, the number and frequency of predominant variants was determined from spike protein mutations and displayed per month according to variant collection date. The mutations are divided onto 3 graphs in which frequencies are up to 100% (1st graph), 8.5% (2nd graph) and 1.75% (3rd graph) for easier visualization. The frequencies are not cumulative but individual representations of each mutation. (TIF) [file ppat.1009772.s001.tif]

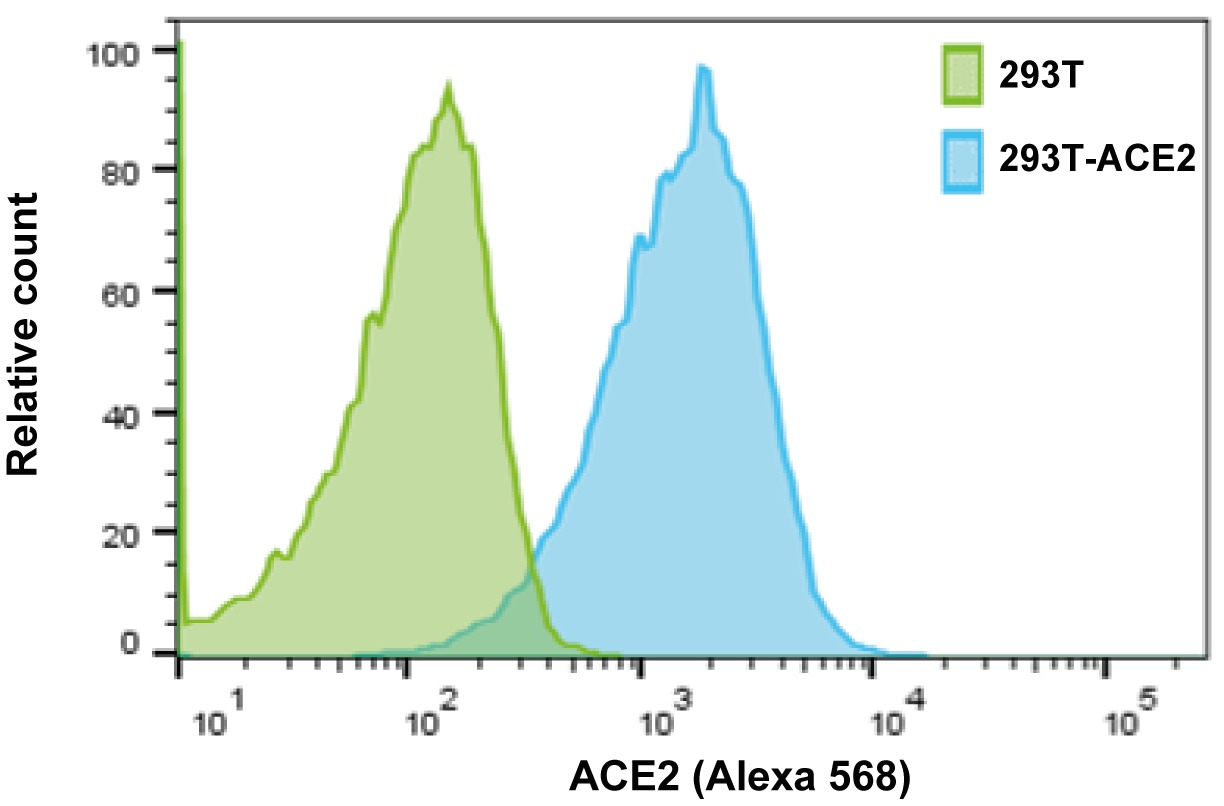

Supplement: S2 Fig — ACE2 expression was measured by flow cytometry, after staining with a goat anti-ACE2 antibody (R&D Systems) followed by staining with an anti-goat antibody conjugated to Alexa 568 (Life Technologies). (TIF) [file ppat.1009772.s002.tif]

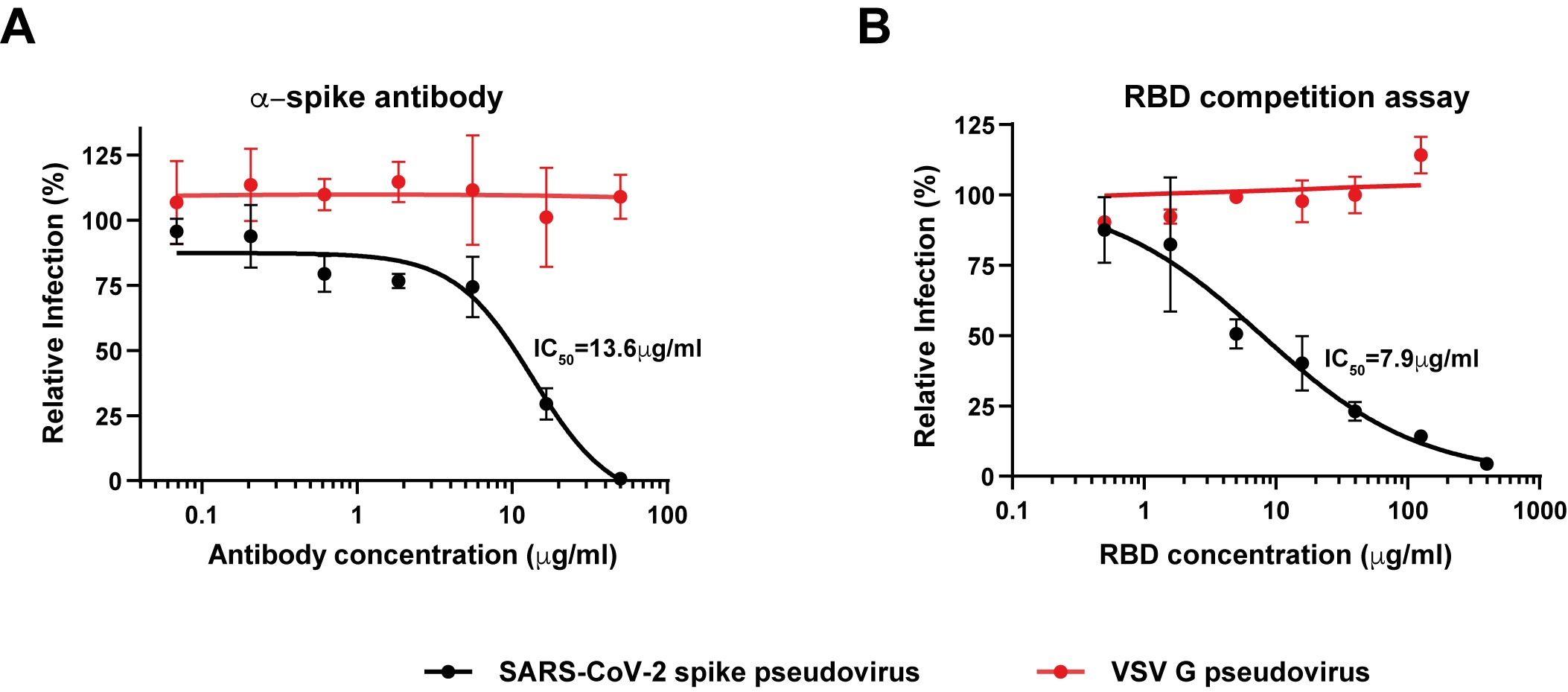

Supplement: S3 Fig — (A) SARS-CoV-2 spike specific antibody was tested for neutralization activity against SARS-CoV-2 spike and vesicular stomatitis virus (VSV) G pseudotyped lentivirus. (B) Neutralization assay of SARS-CoV-2 spike and VSV G pseudoviruses in the presence of spike’s receptor binding domain (RBD). 293T-ACE2 cells were pre-incubated with serial dilutions of RBD before infection with each virus. Half maximal inhibitory concentration (IC50) was calculated for both assays, when possible. (TIF) [file ppat.1009772.s003.tif]

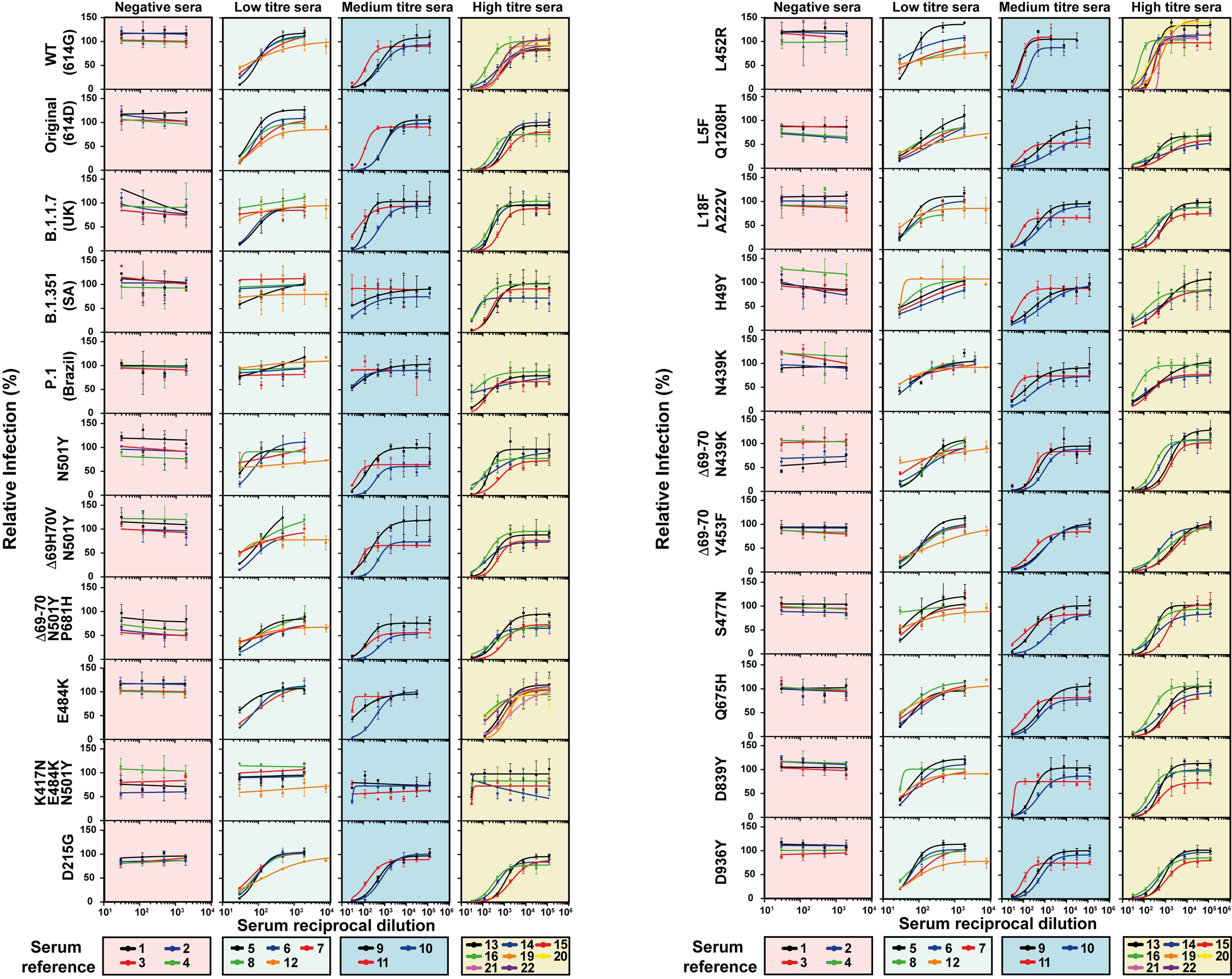

Supplement: S4 Fig — Related to Figs 2 and S5 and S2 Table. Sera from 16–20 individuals were tested for neutralization of WT and 22 viruses. Sera were classified into 4 categories: Negative, Low anti-spike IgG titer (≤1:150), Medium titer (1:450) and High titer (≥1:1350). Triplicates were performed for each tested serum dilution. Error bars represent standard deviation. (TIF) [file ppat.1009772.s004.tif]

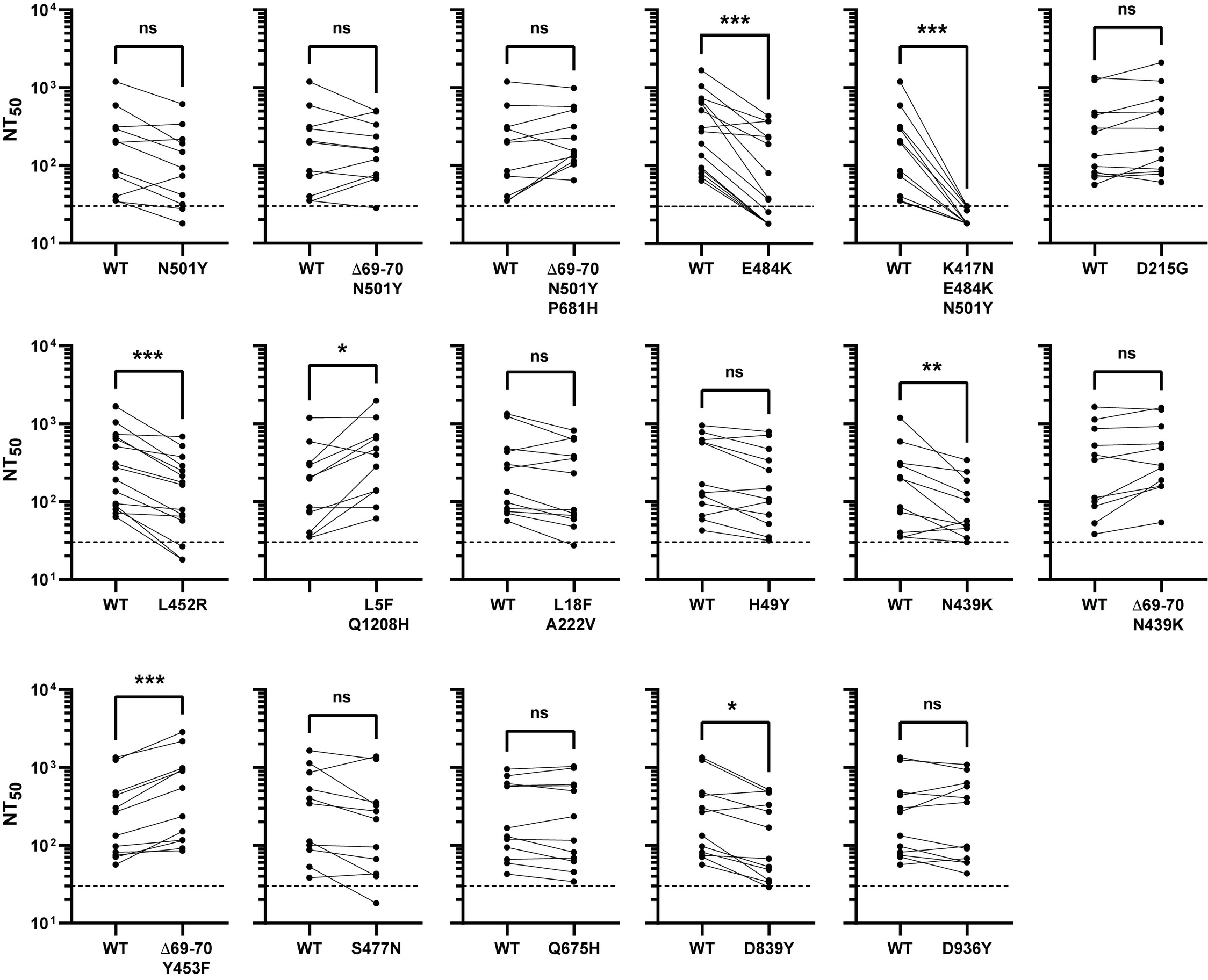

Supplement: S5 Fig — Related to Figs 2 and S4 and S2 Table. Paired analysis of neutralizing activity of each convalescent serum against WT vs mutant virus. NT50 is defined as the inverse of the dilution that achieved a 50% reduction in infection. Dashed lines indicate the limit of detection of the assay (NT50 = 30). ns, non-significant, *p<0.05, **p<0.01, ***p<0.001 by two-tailed Wilcoxon matched-pairs signed-rank test. (TIF) [file ppat.1009772.s005.tif]

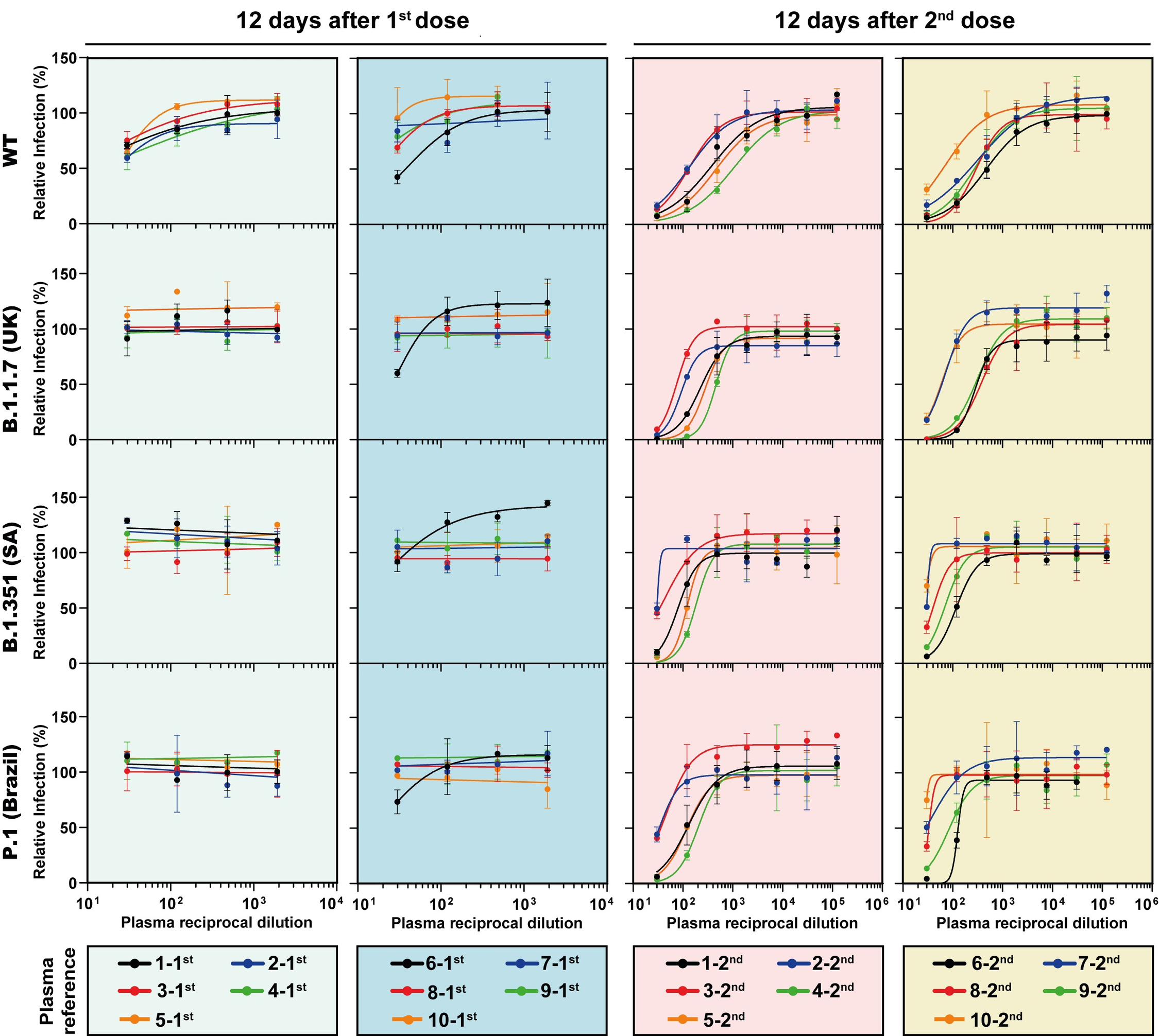

Supplement: S6 Fig — Related to Fig 3 and S3 Table. Plasma was collected from 10 individuals 12 days after the first and the second rounds of vaccination and was tested for neutralization of WT virus and variants of concern. Triplicates were performed for each tested plasma dilution. Error bars represent standard deviation. (TIF) [file ppat.1009772.s006.tif]

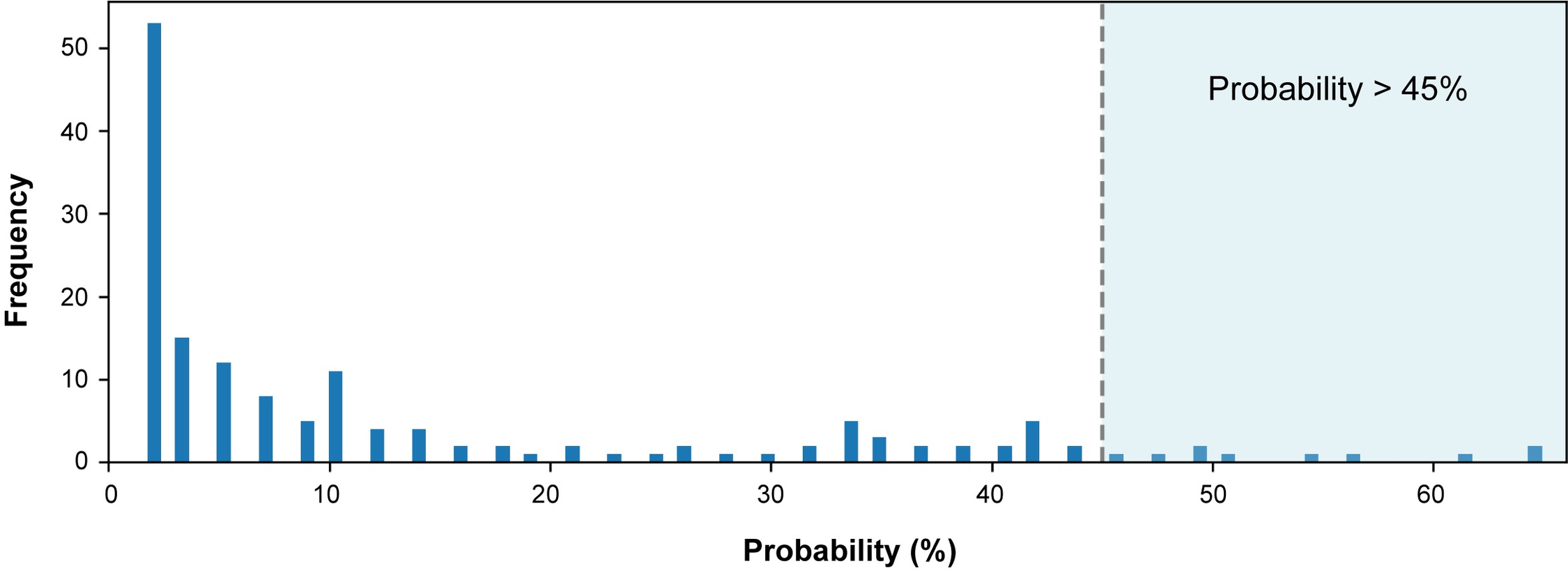

Supplement: S7 Fig — This was computed by dividing the frequency of interaction of spike protein residues with antibodies in 100 bins and calculating the fraction of residues that fall into each bin. (TIF) [file ppat.1009772.s007.tif]

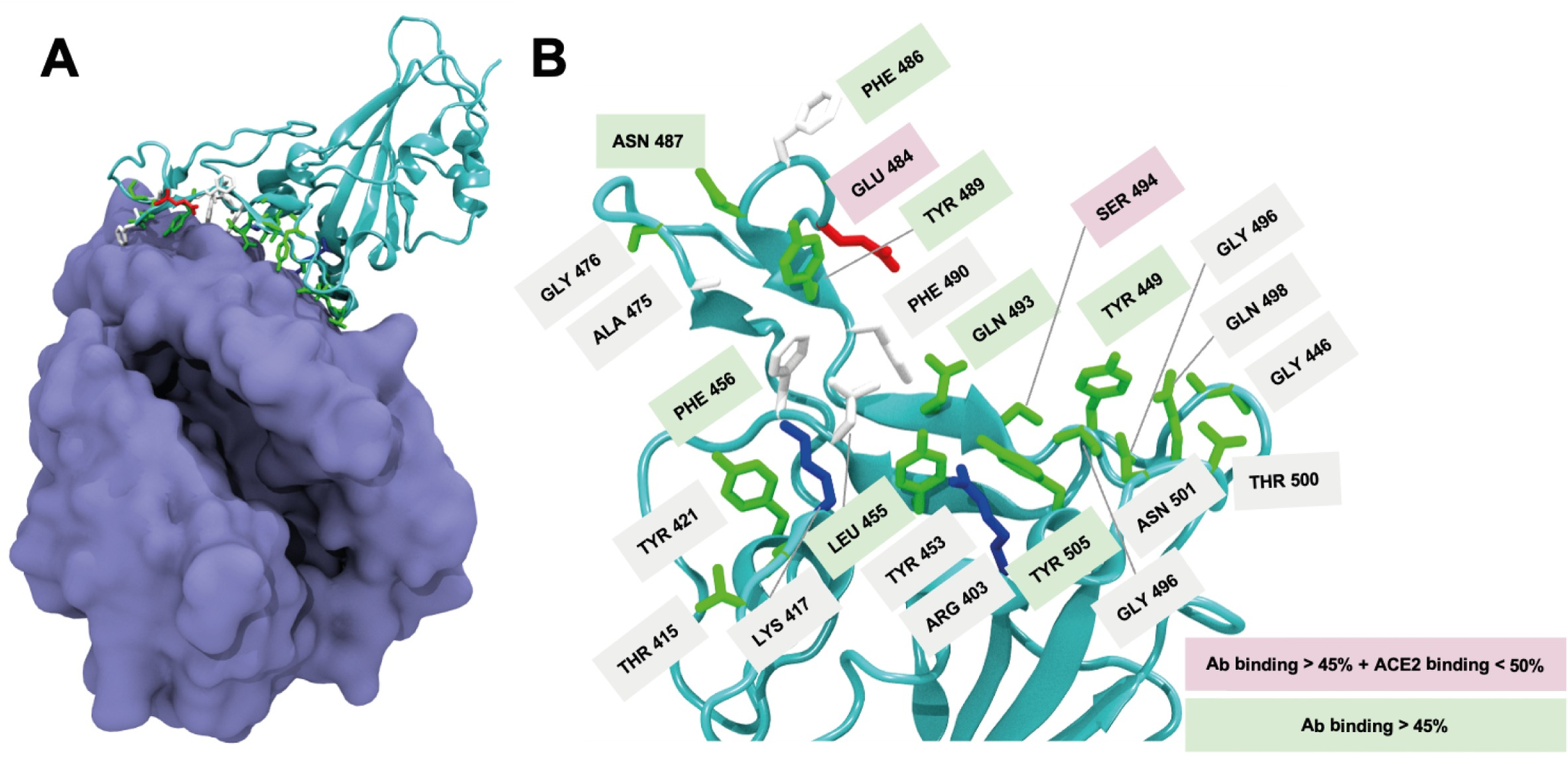

Supplement: S8 Fig — (A) Crystal structure of the SARS-CoV-2 RBD complexed with ACE2. ACE2 is shown as a purple surface and the RBD is shown in a cyan cartoon representation with key antibody interacting residues depicted as sticks. (B) Zoom in to the RBM region of the RBD. Residues relevant for antibody binding (>35% frequency of contact) are depicted as sticks. Of these, the ones with an antibody binding probability higher than 45% have a green label, and those that also have a low frequency of binding to ACE2 (<50%) are labelled in pink. (TIF) [file ppat.1009772.s008.tif]

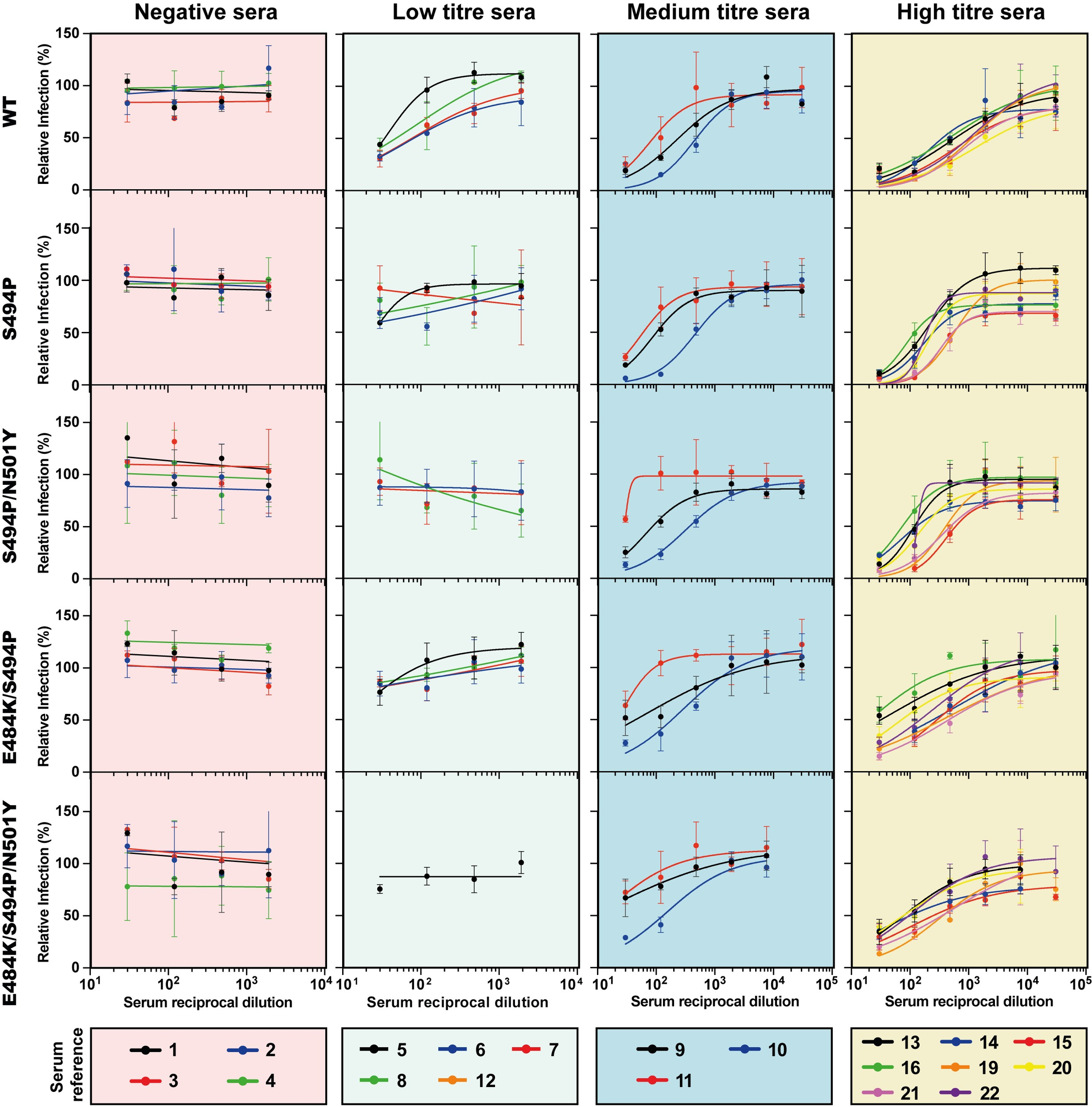

Supplement: S9 Fig — Related to Fig 4 and S5 Table. Sera from 19 individuals were tested for neutralization of WT and S494P mutant viruses. Sera were classified into 4 categories: Negative, Low anti-spike IgG titer (≤1:150), Medium titer (1:450) and High titer (≥1:1350). Triplicates were performed for each tested serum dilution. Error bars represent standard deviation. (TIF) [file ppat.1009772.s009.tif]

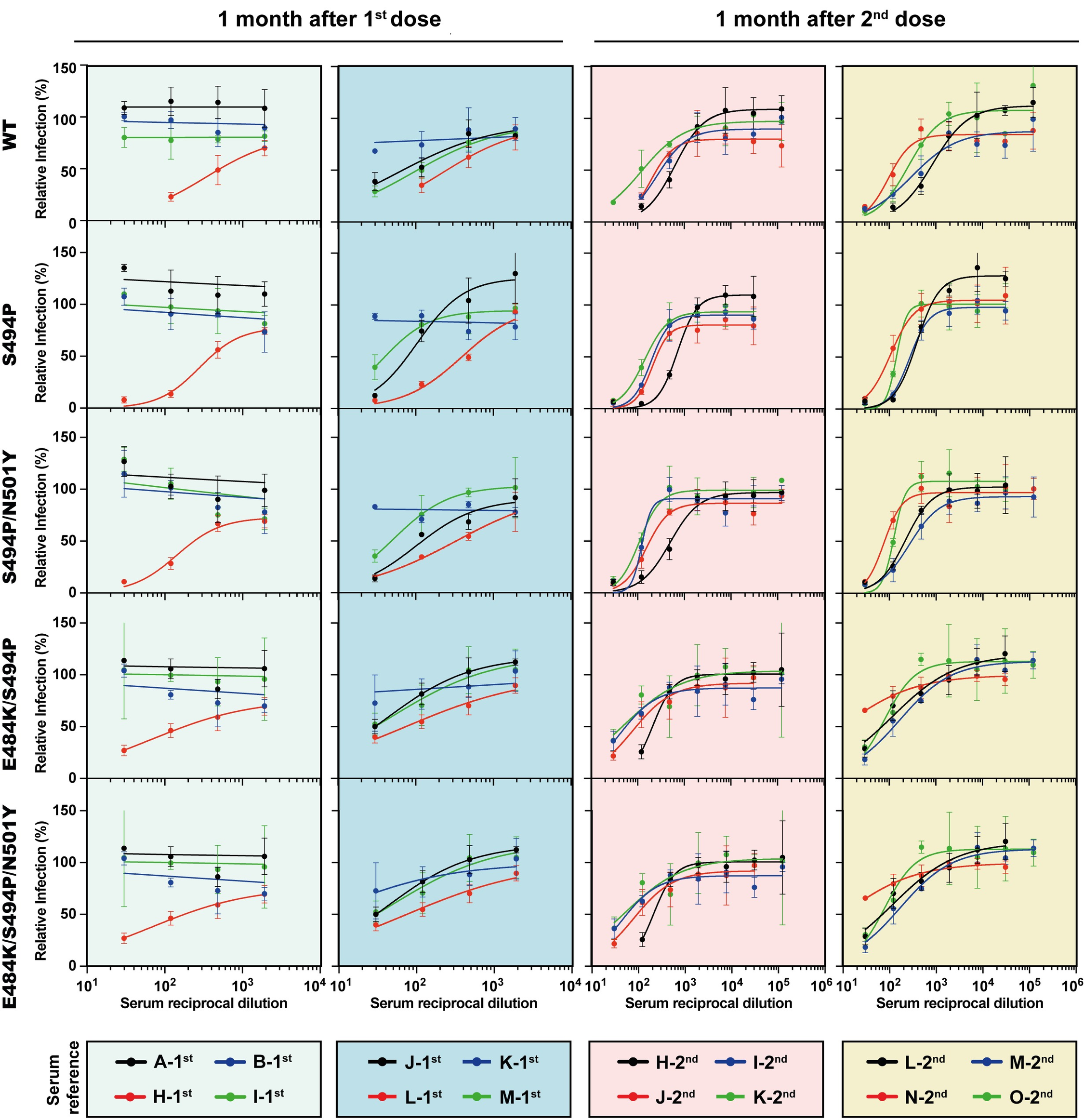

Supplement: S10 Fig — Related to Fig 4 and S6 Table. Serum was collected from 8 individuals 1 month after the first and the second rounds of vaccination, and was tested for neutralization of WT virus and S494P mutants. Triplicates were performed for each tested serum dilution. Error bars represent standard deviation. (TIF) [file ppat.1009772.s010.tif]
