# Supplementary material for: Signatures in SARS-CoV-2 spike protein conferring escape to neutralizing antibodies
Source: PLoS Pathog. 2021 Aug 5;17(8):e1009772. doi: 10.1371/journal.ppat.1009772 (PMC8341613; doi:10.1371/journal.ppat.1009772)
Supplement: S1 Table — Values of current frequency are relative to available sequences of week 11 of 2021 (with a bias corresponding to the efforts that countries/regions deploy to SARS-CoV-2 genome surveillance). (DOCX) [file ppat.1009772.s011.docx]

**S1 Table**. Details of the mutants created and used in this study. Values of current frequency are relative to available sequences of week 11 of 2021 (with a bias corresponding to the efforts that countries/regions deploy to SARS-CoV-2 genome surveillance).

| **#** | **Amino acid mutations** | **References/description** |
| --- | --- | --- |
| 1 | D614G  From the original to the WT | This mutation emerged early in the epidemic and spread rapidly through Europe and North America, becoming dominant. This is the new WT, and all the other mutations are made on this genotype. Several lines of evidence now suggest that SARS-CoV-2 variants carrying this mutation have increased viral entry, infectivity, and transmissibility but not different pathogenicity and do not escape antibody mediated immunity [1-3].  Structural information: D614 Sits in a loop. The mutation to Glycine destroys a salt bridge with Lys 864. It will likely make the loop more mobile. There is a glycan nearby.  FREQUENCY 98.4 % |
| 2 | Δ69H-70V/Δ144/ N501Y/A570D/ P681H/T716I/S982A/  D1118H | Lineage B.1.1.7 (20I/501Y.V1 (UK)) – all defining mutations.  FREQUENCY 65.5% |
| 3 | D80A/D215G/K417/ E484K/N501Y/A701V | Lineage B.1.351/501Y.V2 (South Africa) - all defining mutations.  FREQUENCY 1.9% |
| 4 | L18F/T20N_P26S/ D138Y/R190S/K417T/E484K/N501Y/H655Y/T1027I | Lineage P.1/501Y.V3 (Brazil) - all defining mutations. This lineage was first detected in Manaus in December 2020.  FREQUENCY 0.47% |
| 5 | N501Y | Feature mutation in lineages B.1.1.7 (20I/501Y.V1 (UK)); B.1.351/501Y.V2; P.1/501Y.V3. Amino acid 501 is in the RBD. The N501Y mutation stabilizes virus-binding residues on ACE2 [4,5]. There is convincing evidence that N501Y increases viral transmission [6].  FREQUENCY 71.4% |
| 6 | Δ69H-70V/N501Y | Found in lineage B.1.1.7 (20I/501Y.V1 (UK)) and are considered its most relevant mutations. N501Y was described above. The deletion 69-70 (ΔH69/V70) causes spike gene target failure (SGTF) in some RT-PCR assays [7] and was reported to enhance viral infectivity [8]. Importantly, ΔH69/V70 frequently associates with the mutations N501Y, N439K and Y453F at the RBD, and is also frequently found in association with mutation P681H [8].  FREQUENCY 65.9% |
| 7 | Δ69H-70V/N501Y/ P681H | Found in lineage B.1.1.7 and are considered the principal spike mutations. Has spread to multiple countries and its frequency is increasing. Mutations Δ69H-70V and N501Y were described before. P681H is present in Lineage B.1.1.7 and other circulating variants. It was reported as being able to reduce antibody recognition [9]. In addition, it is localized in an exposed loop (677-688) of significant structural flexibility that is unmodelled in several published structures [8].  FREQUENCY 65.9% |
| 8 | E484K | E484 is in the RBD/RBM and interacts with ACE2 at lysine 31, although with low frequency. It has been associated with antibody escape from therapeutic antibodies, natural infection, convalescent sera, and vaccination-induced immunity [10-12].  FREQUENCY 6.4% |
| 9 | K417N/E484K/N501Y | Mutation present in lineages B.1.351/501Y.V2 (South Africa) (lacks mutations D80A, D215G and A701V) and P.1/501Y.V3 (Brazil) that, in most cases, instead of K417N has the mutation K417T. However, as T and N are similar amino acids, the mutations have similar effects. Deep mutational scanning indicates that N or T change to K in position 417 mutation has minimal effect on spike binding affinity to ACE2 [13]. K417 is a unique ACE2 interacting residue that forms a salt bridge interaction across the central contact region with D30 of ACE2 [5].  FREQUENCY 2.0% |
| 10 | L5F/Q1208H | L5F - Mutations in the N terminal have been shown to affect neutralization [14].  There is no structural information for any of these amino acids because they localize to unstructured regions at the NTD, TD and CD.  FREQUENCY 0.0% |
| 11 | L18F/A222V | L18F – Present in lineage. B.1.1.28.1/P.1, has a mild impact in the structure of spike and protects from some neutralizing monoclonal antibodies [14]. A222V – Mutation that expanded in Europe in lineage B.1.117 and is not associated with antibody escape [14,15]. These mutations are frequently found in association.  FREQUENCY 0.9% |
| 12 | H49Y | Mutation identified in China [16] and not reported to affect the function of spike [3].  FREQUENCY 0.3% |
| 13 | D215G | This mutation is present in lineage B.1.351 and is one of many mutations in the NTD of spike reported to contribute to resistance to neutralizing antibodies [14,17]. Currently, it is found exclusively associated with B. 1.351, however, its prevalence in the past has displayed two peaks unrelated to E484K (S1 Fig).  FREQUENCY 2.2% |
| 14 | N439K | Found in lineages B.1.141 (common in UK in the beginning of pandemics, until June) and B.1.258 (appeared in April, and its presence has been slowly increasing). N439K mutation sits in the RBD, and results show that the virus retains viral fitness but becomes resistant to some neutralizing antibodies [18,19]. N439 sits at the extremity of RBD, but not directly in the contact zone to ACE2. It is under the residues at the contact zone. It is exposed in the "up" conformation of the RBD. In the down conformation it is semi-exposed. A likely zone for antibody binding.  FREQUENCY 2.0% |
| 15 | Δ69H-70V/N439K | Δ69-70 and N439K have been explained individually above. Importantly, ΔH69/V70 frequently associates with the mutations in RBD N501Y, N439K and Y453F [8].  FREQUENCY 2.0% |
| 16 | L452R | Part of lineage B.1.427/9 (USA CAL). It is in RBD and has been shown to increase infectivity [11] and escape antibody binding in a screen in yeast using the RBD and not full-length spike [20,21].  FREQUENCY 4.2% |
| 17 | Δ69H-70V/Y453F | These set of mutations were found together in mink-related infections. They were found initially in Denmark and in the Netherlands and resulted in culling of minks [22]. Δ69-70 was explained above. Y453 is localized in the RBD and was shown to affect neutralization by SARS-CoV-2 specific antibodies [19].  FREQUENCY 0.0% |
| 18 | S477N | Localized at the RBD, was reported to have a modest increase in the affinity of the RBD to ACE2 [13].  FREQUENCY 2.4% |
| 19 | Q675H | Q675H leads to a putative change in glycosylation and was reported to reduce infectivity [11].  FREQUENCY 0.20% |
| 20 | D839Y | Prevalent in Portugal by April 30^th^ (2020) [23]. The aspartic acid 839 is exposed, not making relevant interactions. It may be a zone for antibody targeting. This zone may be important for fusion and/or in the induction of host inflammatory responses.  FREQUENCY 0.01% |
| 21 | D936Y | The aspartic acid 936 is in the fusion core of the heptad repeat 1. It was detected in Sweden and England and was reported to destabilize the post-fusion conformation of spike, while minimally impacting on the stability of the pre-fusion [24]. A distinct paper reported that this mutation increased infectivity but did not affect neutralization by antibodies [25]. This amino acid sits in an exposed helix. Its substitution for a tyrosine appears to be harmless from the perspective of structural stability.  FREQUENCY 0.7% |
| 22 | S494P | The acquisition of mutation S494P was observed for B.1.1.7 lineage at the end of February 2021, and was acquired multiple times, and its frequency is increasing. S494P allows evasion of binding or neutralization by several monoclonal antibodies [26] but has not been shown to impact on neutralization by convalescent or vaccine-induced polyclonal antisera. S494P confers increased binding to ACE2 [13].  FREQUENCY 0.81% |
| 23 | S494P/N501Y | The acquisition of Spike mutation S494P in was observed for B.1.1.7 at the end of February 2021, and was acquired multiple times, and its frequency is increasing.  FREQUENCY 0.44% |
| 24 | E484K/S494P | Although still at a very low frequency, this double mutation is starting to appear.  FREQUENCY 0.01% |
| 25 | E484K/S494P/N501Y | This triple mutation was not detected yet.  FREQUENCY 0.00% |

**References:**

1. Korber B, Fischer WM, Gnanakaran S, Yoon H, Theiler J, Abfalterer W, et al. Tracking Changes in SARS-CoV-2 Spike: Evidence that D614G Increases Infectivity of the COVID-19 Virus. Cell. 2020;182(4):812-27 e19.

2. Volz E, Hill V, McCrone JT, Price A, Jorgensen D, O'Toole A, et al. Evaluating the Effects of SARS-CoV-2 Spike Mutation D614G on Transmissibility and Pathogenicity. Cell. 2021;184(1):64-75 e11.

3. Ozono S, Zhang Y, Ode H, Sano K, Tan TS, Imai K, et al. SARS-CoV-2 D614G spike mutation increases entry efficiency with enhanced ACE2-binding affinity. Nat Commun. 2021;12(1):848.

4. Shang J, Ye G, Shi K, Wan Y, Luo C, Aihara H, et al. Structural basis of receptor recognition by SARS-CoV-2. Nature. 2020;581(7807):221-4.

5. Wang Y, Liu M, Gao J. Enhanced receptor binding of SARS-CoV-2 through networks of hydrogen-bonding and hydrophobic interactions. Proc Natl Acad Sci U S A. 2020;117(25):13967-74.

6. Chen J, Wang R, Wang M, Wei GW. Mutations Strengthened SARS-CoV-2 Infectivity. J Mol Biol. 2020;432(19):5212-26.

7. Vogels CBF, Breban M, Alpert T, Petrone ME, Watkins AE, Hodcroft EB, et al. PCR assay to enhance global surveillance for SARS-CoV-2 variants of concern. medRxiv 2021.

8. Kemp S, Meng B, Ferreira I, Datir R, Harvey W, Collier D, et al. Recurrent emergence and transmission of a SARS-CoV-2 spike deletion H69/V70. bioRxiv. 2021.

9. Haynes WA, Kamath K, Lucas C, Shon J, Iwasaki A. Impact of B.1.1.7 variant mutations on antibody recognition of linear SARS-CoV-2 epitopes. medRxiv. 2021.

10. Xie X, Liu Y, Liu J, Zhang X, Zou J, Fontes-Garfias CR, et al. Neutralization of SARS-CoV-2 spike 69/70 deletion, E484K and N501Y variants by BNT162b2 vaccine-elicited sera. Nat Med. 2021.

11. Li Q, Wu J, Nie J, Zhang L, Hao H, Liu S, et al. The Impact of Mutations in SARS-CoV-2 Spike on Viral Infectivity and Antigenicity. Cell. 2020;182(5):1284-94 e9.

12. Sabino EC, Buss LF, Carvalho MPS, Prete CA, Crispim MAE, Fraiji NA, et al. Resurgence of COVID-19 in Manaus, Brazil, despite high seroprevalence. The Lancet. 2021;397(10273):452-5.

13. Starr TN, Greaney AJ, Hilton SK, Ellis D, Crawford KHD, Dingens AS, et al. Deep Mutational Scanning of SARS-CoV-2 Receptor Binding Domain Reveals Constraints on Folding and ACE2 Binding. Cell. 2020;182(5):1295-310 e20.

14. McCallum M, Marco A, Lempp F, Tortorici MA, Pinto D, Walls AC, et al. N-terminal domain antigenic mapping reveals a site of vulnerability for SARS-CoV-2. bioRxiv. 2021.

15. Hodcroft EB, Zuber M, Nadeau S, Crawford KHD, Bloom JD, Veesler D, et al. Emergence and spread of a SARS-CoV-2 variant through Europe in the summer of 2020. medRxiv. 2020.

16. Wei X, Li X, Cui J. Evolutionary perspectives on novel coronaviruses identified in pneumonia cases in China. Natl Sci Rev. 2020;7(2):239-42.

17. Wibmer CK, Ayres F, Hermanus T, Madzivhandila M, Kgagudi P, Oosthuysen B, et al. SARS-CoV-2 501Y.V2 escapes neutralization by South African COVID-19 donor plasma. bioRxiv. 2021.

18. Thomson EC, Rosen LE, Shepherd JG, Spreafico R, da Silva Filipe A, Wojcechowskyj JA, et al. Circulating SARS-CoV-2 spike N439K variants maintain fitness while evading antibody-mediated immunity. Cell. 2021;184(5):1171-87 e20.

19. Baum A, Fulton BO, Wloga E, Copin R, Pascal KE, Russo V, et al. Antibody cocktail to SARS-CoV-2 spike protein prevents rapid mutational escape seen with individual antibodies. Science. 2020;369(6506):1014-8.

20. Greaney AJ, Starr TN, Gilchuk P, Zost SJ, Binshtein E, Loes AN, et al. Complete Mapping of Mutations to the SARS-CoV-2 Spike Receptor-Binding Domain that Escape Antibody Recognition. Cell Host Microbe. 2021;29(1):44-57 e9.

21. McCallum M, Bassi J, De Marco A, Chen A, Walls AC, Di Iulio J, et al. SARS-CoV-2 immune evasion by variant B.1.427/B.1.429. BioRxiv. 2021.

22. van Dorp L, Tan CCS, Lam SD, Richard D, Owen C, Berchtold D, et al. Recurrent mutations in SARS-CoV-2 genomes isolated from mink point to rapid host-adaptation. bioRxiv 2020.

23. Borges V, Isidro J, Cortes-Martins H, Duarte S, Vieira L, Leite R, et al. 1On the track of the D839Y mutation in the SARS-CoV-2 Spike fusion peptide: emergence and geotemporal spread of a highly prevalent variant in Portugal. medRxiv. 2020.

24. Cavallo L, Oliva R. D936Y and Other Mutations in the Fusion Core of the SARS-Cov-2Spike Protein Heptad Repeat 1 Undermine the Post-Fusion Assembly. bioRxiv. 2020.

25. Stukalov A, Girault V, Grass V, Bergant V, Karayel O, Urban C, et al. Multi-level proteomics reveals host-perturbation strategies of SARS-CoV-2 and SARS-CoV. bioRxiv. 2020.

26. Liu Z, VanBlargan LA, Bloyet L-M, Rothlauf PW, Chen RE, Stumpf S, et al. Landscape analysis of escape variants identifies SARS-CoV-2 spike mutations that attenuate monoclonal and serum antibody neutralization. bioRxiv. 2021:2020.11.06.372037.
