# Supplementary material for: Signatures in SARS-CoV-2 spike protein conferring escape to neutralizing antibodies
Source: PLoS Pathog. 2021 Aug 5;17(8):e1009772. doi: 10.1371/journal.ppat.1009772 (PMC8341613; doi:10.1371/journal.ppat.1009772)
Supplement: S2 Table — (DOCX) [file ppat.1009772.s012.docx]

**S2 Table.** IgG antibody titers against SARS-CoV-2 spike protein and neutralizing titers (NT_50_) of convalescent sera against WT and mutant spike pseudoviruses.

| **Serum ID** | **ELISA IgG titer** | | **NT_50_ (95% confidence interval)** | | | | | | | | | | | |
| --- | --- | --- | --- | --- | --- | --- | --- | --- | --- | --- | --- | --- | --- | --- |
|  |  |  | **WT**  **(614G)** | **Original (614D)** | **B.1.1.7**  **(U.K.)** | **B.1.351**  **(S.A.)** | **P.1**  **(Brazil)** | **N501Y** | **Δ69-70**  **N501Y** | **Δ69-70/N501Y P681H** | **E484K** | **K417N/E484K N501Y** | **L452R** | **S494P** |
| 1* | Negative control | <50 | <30 | <30 | <30 | <30 | <30 | <30 | <30 | <30 | <30 | <30 | <30 | <30 |
| 2 |  | <50 | <30 | <30 | <30 | <30 | <30 | <30 | <30 | <30 | <30 | <30 | <30 | <30 |
| 3 |  | <50 | <30 | <30 | <30 | <30 | <30 | <30 | <30 | <30 | <30 | <30 | <30 | <30 |
| 4 |  | <50 | <30 | <30 | <30 | <30 | <30 | <30 | <30 | <30 | <30 | <30 | <30 | <30 |
| 5 | Low titer | 50 | 97 (83-114) | 57 (48-68) | 84 (41-545) | <30 | <30 | 32 (?-?) | 120 (34-?) | 64 (37-304) | <30 | <30 | 65 (52-80) | <30 |
| 6 |  | 150 | 74 (51-119) | 71 (61-82) | 70 (48-102) | <30 | <30 | 73 (55-99) | 77 (41-?) | 103 (42-?) | <30 | <30 | <30 | <30 |
| 7 |  | 150 | 81 (41-128 | 87 (59-184) | <30 | <30 | <30 | 30 (?-?) | <30 | 114 (16-?) | <30 | <30 | <30 | <30 |
| 8 |  | 150 | 71 (53-102) | 53 (42-68) | <30 | <30 | <30 | <30 | 68 (25-?) | 141 (60-283) | <30 | <30 | <30 | <30 |
| 12 |  | 150 | 43 (19-94) | 76 (55-105) | <30 | <30 | <30 | <30 | <30 | <30 | n.d. | <30 | <30 | <30 |
| 9 | Medium titer | 450 | 573 (242-3770) | 924 (696-1250) | 123 (75-204) | <30 | <30 | 149 (75-310) | 235 (118-522) | 153 (101-236) | 37 (14-115) | <30 | 68 (44-?) | 55 (39-77) |
| 10 |  | 450 | 621 (407-1019) | 854 (677-1105) | 527 (396-720) | 41 (10-556) | <30 | 339 (166-744) | 492 (357-696) | 520 (365-797) | 372 (264-545) | <30 | 177 (?-258) | 154 (122-199) |
| 11 |  | 450 | 120 (92-152) | 117 (101-135) | 53 (33-95) | <30 | <30 | 42 (26-69) | 69 (51-?) | 130 (74-254) | <30 | <30 | 79 (47-?) | 30 (28-35) |
| 13 | Hig**h** titer | 4050 | 954 (574-1606) | 946 (762-1180) | 241 (?-?) | 339 (183-589) | 161 (80-345) | 192 (122-340) | 333 (204-536) | 572 (430-776) | 188 (126-312) | 30 (?-?) | 376 (308-?) | 293 (213-408) |
| 14 |  | 4050 | 581 (278-1674) | 769 (576-1052) | 283 (170-454) | 40 (24-70) | <30 | 217 (107-761) | 159 (95-312) | 226 (127-440) | 236 (159-388) | <30 | 165 (?-201) | 131 (87-209) |
| 15 |  | 4050 | 783 (655-944) | 1248 (855-1859) | 663 (471-1006) | 226 (136-388) | 137 (80-218) | 613 (351-1579) | 504 (374-715) | 984 (590-1660) | 229 (166-330) | 30 (24-37) | 288 (204-?) | 397 (281-597) |
| 16 |  | 1350 | 167 (120-235) | 263 (159-433) | 217 (167-281) | 66 (31-125) | 32 (2-1.1x10^6^) | 93 (62-141) | 162 (118-223) | 315 159-1052) | <30 | <30 | 57 | 60 (40-89) |
| 19 |  | 4050 | 729 (?-1075) | n.d. | n.d. | n.d. | n.d. | n.d. | n.d. | n.d. | 371 (245-553) | n.d. | 686 (503-982) | 345 (259-482) |
| 20 |  | 1350 | 641 (528-789) | n.d. | n.d. | n.d. | n.d. | n.d. | n.d. | n.d. | 38 (6-2,9x10^7^) | n.d. | 250 (177-355) | 283 (209-390) |
| 21 |  | 1350 | 1668 (124-2333) | n.d. | n.d. | n.d. | n.d. | n.d. | n.d. | n.d. | 435 (261-1002) | n.d. | 518 (?-735) | 653 (399-1238) |
| 22 |  | 1350 | 684 (591-797) | n.d. | n.d. | n.d. | n.d. | n.d. | n.d. | n.d. | 80 (26-2727) | n.d. | 213 (144-314) | 261 (173-404) |
| **Serum ID** | **ELISA IgG titer** | | **L5F/Q1208H**  **S1252P** | **L18F**  **A222V** | **H49Y** | **D215G** | **Δ69-70**  **N439K** | **N439K** | **L452R** | **Δ69-70**  **Y453F** | **S477N** | **Q675H** | **D839Y** | **D936Y** |
| 1* | Negative control | <50 | <30 | <30 | <30 | <30 | <30 | <30 | <30 | <30 | <30 | <30 | <30 | <30 |
| 2 |  | <50 | <30 | <30 | <30 | <30 | <30 | <30 | <30 | <30 | <30 | <30 | <30 | <30 |
| 3 |  | <50 | <30 | <30 | <30 | <30 | <30 | <30 | <30 | <30 | <30 | <30 | <30 | <30 |
| 4 |  | <50 | <30 | <30 | <30 | <30 | <30 | <30 | <30 | <30 | <30 | <30 | <30 | <30 |
| 5 | Low titer | 50 | 138 (47-?) | 59 (46-76) | 68 (25-?) | 90 (77-103) | 158 (114-248) | 50 (10-120) | 60 (58-62) | 117 (106-129) | 40 (5-?) | 62 (39-121) | 53 (44-64) | 60 (51-71) |
| 6 |  | 150 | 282 (118-1x10^5^) | 66 (37-351) | 148 (31-?) | 84 (70-99) | 155 (105-225) | 45 (13-?) | <30 | 92 (59-196) | 66 (38-161) | 82 (51-207) | 67 (56-83) | 60 (47-75) |
| 7 |  | 150 | 140 (45-?) | 78 (38-138) | 100 (36-?) | 61 (54-68) | 54 (15-?) | <30 | 37 (2-?) | 85 (66-119) | 43 (12-93) | 69 (33-40536) | 49 (25-7.4x10^5^) | 97 (68-168) |
| 8 |  | 150 | 61 (19-?) | 48 (36-68) | 35 (0-?) | 77 (55-114) | 189 (100-2444) | 56 (5-?) | <30 | 115 (77-237) | <30 | 45 (29-127) | <30 | 43 (33-60) |
| 12 |  | 150 | 78 (9-?) | <30 | 32 (27-47) | 120 (85-176) | <30 | <30 | 168 (124-243) | 150 (98-256) | 17 (1-44) | 34 (7-341) | 34 (17-53) | 68 (44-103) |
| 9 | Medium titer | 450 | 699 (377-1559) | 384 (296-511) | 253 (145-539) | 484 (382-628) | 487 (286-825) | 127 (54-1170) | 307 (213-418) | 982 (698-1474) | 274 (162-590) | 584 (333-1281) | 270 (180-401) | 406 (330-504) |
| 10 |  | 450 | 1973 (820-8651) | 662 (418-1120) | 715 (288-5790) | 721 (561-941) | 926 (749-1149) | 243 (134-608) | 67 (44-?) | 907 (615-1484) | 1379(1044-1893) | 500 (312-872) | 496 (382-654) | 631 (478-852) |
| 11 |  | 450 | 85 (51-151) | 70 (50-?) | 52 (31-85) | 161 (118-228) | 272 (185-?) | 34 (20-52) | <30 | 235 (171-339) | 95 (54-184) | 116 (72-221) | 35 (?-63) | 91 (65-119) |
| 16 | Hig**h** titer | 1350 | 477 (199-2315) | 231 (160-344) | 107 (17-5582) | 300 (207-451) | 290 (233-359) | 342 (194-765) | 538 (404-720) | 941 (535-2061) | 216 (161-298) | 236 (149-377) | 170 (99-292) | 936 (786-1116) |
| 13 |  | 4050 | 399 (285-558) | 825 (671-1023) | 793 (421-2152) | 1212 (1057-1397) | 1609 (1212-2213) | 105 (45-1015) | 167 (109-268) | 2170 (1585-3170) | 328 (277-383) | 1032 (738-1453) | 520 (431-628) | 570 (466-706) |
| 14 |  | 4050 | 648 (194-2x10^6^) | 358 (271-481) | 339 (81-1.6x10^7^) | 506 (395-662) | 552 (366-878) | 186 (129-275) | 581 (325-1064) | 544 (402-786) | 353 (211-682) | 603 (340-1321) | 331 (262-421) | 1082 (869-1353) |
| 15 |  | 4050 | 1208 (741-2273) | 627 (500-795) | 474 (136-3404) | 2094 (1578-2919) | 1520 (1150-2010) | 45 (27-73) | 135 (68-279) | 2858 (2013-4425) | 1276 (863-1959) | 987 (657-1947) | 475 278-871) | 357 (234-567) |

* pre-pandemic pool ? - could not be calculated n.d. – not done
