# Supplementary material for: Signatures in SARS-CoV-2 spike protein conferring escape to neutralizing antibodies
Source: PLoS Pathog. 2021 Aug 5;17(8):e1009772. doi: 10.1371/journal.ppat.1009772 (PMC8341613; doi:10.1371/journal.ppat.1009772)
Supplement: S3 Table — (DOCX) [file ppat.1009772.s013.docx]

**S3 Table.** IgG antibody titers against SARS-CoV-2 spike protein and neutralizing titers (NT_50_) against WT and variant pseudoviruses of plasma from vaccinated individuals, collected 12 days after the first and after the second doses of the vaccine.

| **Plasma ID** | **ELISA IgG titer** | **NT_50_ (95% confidence interval)** | | | |
| --- | --- | --- | --- | --- | --- |
|  |  | **WT** | **B.1.1.7 (UK)** | **B.1.351 (SA)** | **P.1 (Brazil)** |
| v1-1^st^ | 4050 | <30 | <30 | <30 | <30 |
| v2-1^st^ | 4050 | <30 | <30 | <30 | <30 |
| v3-1^st^ | 1350 | <30 | <30 | <30 | <30 |
| v4-1^st^ | 4050 | <30 | <30 | <30 | <30 |
| v5-1^st^ | 4050 | <30 | <30 | <30 | <30 |
| v6-1^st^ | 12150 | 40 (24-68) | 31 (21-43) | <30 | <30 |
| v7-1^st^ | 4050 | <30 | <30 | <30 | <30 |
| v8-1^st^ | 1350 | <30 | <30 | <30 | <30 |
| v9-1^st^ | 1350 | <30 | <30 | <30 | <30 |
| v10-1^st^ | 450 | <30 | <30 | <30 | <30 |
| v1-2^nd^ | 36450 | 380 (210 -952) | 224 (158-324) | 79 (52-112) | 131 (87-209) |
| v2-2^nd^ | 36450 | 134 (84-222) | 92 (70-?) | 30 (24-38) | 33 (19-49) |
| v3-2^nd^ | 109350 | 131 (104 -166) | 75 (59-?) | 43 (29-63) | 46 (35-61) |
| v4-2^nd^ | 36450 | 1053 (777-1486) | 458 (402 -515) | 185 (?-262) | 203 (?-320) |
| v5-2^nd^ | 36450 | 457 (288 -790) | 277 (227-333) | 122 (92-159) | 117 (90-157) |
| v6-2^nd^ | 109350 | 456 (324-663) | 279 (197-?) | 116 (94-143) | 125 (113 -164) |
| v7-2^nd^ | 36450 | 316 (194-536) | 71 (54-91) | 30 (26-38) | 35 (17-56) |
| v8-2^nd^ | 36450 | 290 (189-439) | 381 (285 -539) | 40 (26-67) | 33 (?-56) |
| v9-2^nd^ | 109350 | 291 (190-460) | 310 (234-409) | 71 (52-95) | 83 (57-120) |
| v10-2^nd^ | 136450 | 72 (47-109) | 61 (42-85) | <30 | <30 |

? - could not be calculated
