# Supplementary material for: Signatures in SARS-CoV-2 spike protein conferring escape to neutralizing antibodies
Source: PLoS Pathog. 2021 Aug 5;17(8):e1009772. doi: 10.1371/journal.ppat.1009772 (PMC8341613; doi:10.1371/journal.ppat.1009772)
Supplement: S4 Table — These antibodies were chosen due to the availability of their structure resolved together with the spike (or just the RBD region) in the PDB repository [94]. (DOCX) [file ppat.1009772.s014.docx]

**S4 Table.** Summary of antibodies studied in the spike-antibody complexes. These antibodies were chosen due to the availability of their structure resolved together with spike (or just the RBD region) in the PDB repository [94].

| **SARS-CoV-2 antibodies studied** | | | | | | |
| --- | --- | --- | --- | --- | --- | --- |
| 15033 | 2-4 | 2-15 | 15033-7 | 2G12 | 2H2 | 3C1 |
| 4A8 | B38 | BD-236 | BD-368-2 | BD-604 | BD-629 | BD23 |
| C002 | C102 | C104 | C105 | C110 | C119 | C121 |
| C135 | C144 | C1A-B12 | C1A-B3 | C1A-C2 | C1A-F10 | CB6 |
| CV30 | DH1047 | DH1050.1 | EY6A | FC05 | H014 | LY-CoV555 |
| CC12.3 | COVA1-16 | COVA2-04 | COVA2-39 | CT-P59 | CV07-250 | CV07-270 |
| P17 | P2B-2F6 | P2C-1A3 | P2C-1F11 | REGN10933 | REGN10987 | S2A4 |
| S2E12 | S2H13 | S2H14 | S2M11 | S304 | S309 | STE90-C11 |
| No name  (7cjf) |  | | | | | |
