# Supplementary material for: Signatures in SARS-CoV-2 spike protein conferring escape to neutralizing antibodies
Source: PLoS Pathog. 2021 Aug 5;17(8):e1009772. doi: 10.1371/journal.ppat.1009772 (PMC8341613; doi:10.1371/journal.ppat.1009772)
Supplement: S5 Table — (DOCX) [file ppat.1009772.s015.docx]

**S5 Table.** IgG antibody titers against SARS-CoV-2 spike protein and neutralizing titers (NT_50_) of convalescent sera against WT and mutant spike pseudoviruses.

| **Serum ID** | **ELISA IgG titer** | | **NT_50_ (95% confidence interval)** | | | | |
| --- | --- | --- | --- | --- | --- | --- | --- |
|  |  |  | **WT** | **S494P** | **S494P/N501Y** | **E484K/S494P** | **E484K/S494P/N501Y** |
| 1* | Negative control | <50 | <30 | <30 | <30 | <30 | <30 |
| 2 |  | <50 | <30 | <30 | <30 | <30 | <30 |
| 3 |  | <50 | <30 | <30 | <30 | <30 | <30 |
| 4 |  | <50 | <30 | <30 | <30 | <30 | <30 |
| 5 | Low titer | 50 | 39 (30-53) | <30 | <30 | <30 | <30 |
| 6 |  | 150 | 64 (19-?) | <30 | <30 | <30 | n.d. |
| 7 |  | 150 | 80 (38-?) | <30 | <30 | <30 | n.d. |
| 8 |  | 150 | 89 (33-?) | <30 | <30 | <30 | n.d. |
| 9 | Medium titer | 450 | 226 (128-408) | 86 (53-139) | 67 (48-92) | 73 (10-?) | <30 |
| 10 |  | 450 | 448 (260-754) | 459 (353 -626) | 317 (236-439) | 284 (129-1066) | 154 (79-1014) |
| 11 |  | 450 | 77 (29-187) | 55 (26-121) | <30 | <30 | <30 |
| 13 | Hig**h** titer | 4050 | 491 (243-2215) | 206 (149-289) | 112 (76-161) | 49 (11-1.3x10^7^) | 77 (37-350) |
| 14 |  | 4050 | 211 (90-503) | 86 (53-139) | 70 (49-100) | 243 (111-429) | 39 (17-4197) |
| 15 |  | 4050 | 579 (350-1026) | 459 (353 -626) | 405 (286-574) | 277 (150-2969) | 106 (35-1407) |
| 16 |  | 1350 | 503 (158-1.4x10^6^) | 55 (26-121) | 71 (38-128) | <30 | n.d. |
| 19 |  | 4050 | 1214 (618-7241) | 206 (149-289) | 374 (206-635) | 340 (198-577) | 313 (148-797) |
| 20 |  | 1350 | 1324 (593-16341) | 168 (111-273) | 139 (96-190) | 56 (23-180) | 68 (25-4931) |
| 21 |  | 1350 | 822 (487-2417) | 329 (258-410) | 342 (231-504) | 415 (149-8.6x10^4^) | 322 (152-654) |
| 22 |  | 1350 | 1332 (580-19344) | 84 (62-112) | 130 (?-211) | 250 (92-7.4x10^5^) | 113 (32-2370) |

* pre-pandemic pool n.d. – not done ? - could not be calculated
