# Supplementary material for: Signatures in SARS-CoV-2 spike protein conferring escape to neutralizing antibodies
Source: PLoS Pathog. 2021 Aug 5;17(8):e1009772. doi: 10.1371/journal.ppat.1009772 (PMC8341613; doi:10.1371/journal.ppat.1009772)
Supplement: S6 Table — (DOCX) [file ppat.1009772.s016.docx]

**S6 Table.** IgG antibody titers against SARS-CoV-2 spike protein and neutralizing titers (NT_50_) against WT and mutant pseudoviruses of serum from vaccinated individuals, collected 1 month after the first and after the second doses of the vaccine.

| **Serum ID** | **ELISA IgG titer** | **NT_50_ (95% confidence interval)** | | | | |
| --- | --- | --- | --- | --- | --- | --- |
|  |  | **WT** | **S494P** | **S494P/N501Y** | **E484K/S494P** | **E484K/S494P/N501Y** |
| A-1^st^ | 150 | <30 | <30 | <30 | <30 | <30 |
| B-1^st^ | 450 | <30 | <30 | <30 | <30 | <30 |
| H-1^st^ | 36450 | 336 (116-?) | 278 (202-452) | 142 (99-229) | 284 (43-?) | 70 (25-?) |
| I-1^st^ | 4050 | <30 | <30 | <30 | <30 | <30 |
| J-1^st^ | 12150 | 60 (18-?) | 105 (56-1115) | 102 (50-1.5X10^5^) | 52 (16-105) | 44 (23-1364) |
| K-1^st^ | 1350 | <30 | <30 | <30 | <30 | <30 |
| L-1^st^ | 12150 | 219 (74-?) | 395 (307-505) | 332 (219-509) | 233 (42-?) | 74 (43-114) |
| M-1^st^ | 36450 | 91 (51-819) | 37 (22-69) | 51 (22-19545) | 123 (36-?) | 49 (5-?) |
| H-2^nd^ | 328050 | 631 (422-948) | 715 (?-909) | 495 (345-697) | 282 (143-575) | 210 (136-331) |
| I-2^nd^ | 12150 | 263 (164-415) | 192 (?-251) | 122 (84-154) | 128 (57-746) | 43 (18-79) |
| J-2^nd^ | 36450 | 191 (116-313) | 206 (?-292) | 154 (116-206) | 63 (19-1158) | 78 (39-221) |
| K-2^nd^ | 36450 | 119 (70-213) | 140 (97-205) | 109 (74-151) | 69 (30-139) | 52 (0-?) |
| L-2^nd^ | 36450 | 858 (556-1430) | 389 (300-517) | 221 (143-349) | 138 (57-462) | 118 (53-1860) |
| M-2^nd^ | 36450 | 301 (122-2023) | 270 (202-353) | 264 (172-411) | 111 (59-220) | 170 (106-289) |
| N-2^nd^ | 12150 | 97 (63-138) | 106 (70-164) | 77 (52-?) | 34 (21-49) | <30 |
| O-2^nd^ | 36450 | 259 (125-670) | 142 (120-209) | 126 (96-162) | 77 (39-147) | 79 (38-146) |

? - could not be calculated
