# Supplementary material for: Signatures in SARS-CoV-2 spike protein conferring escape to neutralizing antibodies
Source: PLoS Pathog. 2021 Aug 5;17(8):e1009772. doi: 10.1371/journal.ppat.1009772 (PMC8341613; doi:10.1371/journal.ppat.1009772)
Supplement: S7 Table — (DOCX) [file ppat.1009772.s017.docx]

**S7 Table.** Primers used in this study.

| **Primer name** | **Sequence** |
| --- | --- |
| S_trunc__Forward | 5’-TCGAACCGGTTCAACGGCATCGGA-3’ |
| S_trunc__Reverse | 5’-TATAGAGCTCTCAGCAGCAGCTGCCACAGCTAC-3’ |
| Spike_D614G_Fw | 5’-CAGTGCTGTACCAGGGCGTGAACTGTACCGA-3’ |
| Spike_D614G_Rv | 5’-TCGGTACAGTTCACGCCCTGGTACAGCACTG-3’ |
| Spike_L5F_Fw | 5’-CCACCATGTTCGTGTTTTTCGTGCTGCTGCCTCTG-3’ |
| Spike_L5F_Rv | 5’-CAGAGGCAGCAGCACGAAAAACACGAACATGGTGG-3’ |
| Spike_L18F_Fw | 5’- CAGCCAGTGTGTGAACTTCACCACAAGAACCCAGC-3’ |
| Spike_L18F_Rv | 5’- GCTGGGTTCTTGTGGTGAAGTTCACACACTGGCTG-3’ |
| Spike_T20N_Fw | 5’-CAGTGTGTGAACCTGACCAATAGAACCCAGCTGCCTC-3’ |
| Spike_T20N_Rv | 5’-GAGGCAGCTGGGTTCTATTGGTCAGGTTCACACACTG-3’ |
| Spike_P26S_Fw | 5’-GAACCCAGCTGCCTTCAGCCTACACCAAC-3’ |
| Spike_P26S_Rv | 5’-GTTGGTGTAGGCTGAAGGCAGCTGGGTTC-3’ |
| Spike_H49Y_Fw | 5’-CAGATCCAGCGTGCTGTATTCTACCCAGGACCTGT-3’ |
| Spike_H49Y_Rv | 5’-ACAGGTCCTGGGTAGAATACAGCACGCTGGATCTG-3’ |
| Spike_Δ69-70_Fw | 5’-GGTTCCACGCCATCTCCGGCACCAATGG-3’ |
| Spike_Δ69-70_Rv | 5’-CCATTGGTGCCGGAGATGGCGTGGAACC-3’ |
| Spike_D80A_Fw | 5’-CACCAAGAGATTCGCCAACCCCGTGCTGC-3’ |
| Spike_D80A_Rv | 5’-GCAGCACGGGGTTGGCGAATCTCTTGGTG-3’ |
| Spike_D138Y_Fw | 5’-GTTCCAGTTCTGCAACTATCCCTTCCTGGGCGTCT-3’ |
| Spike_D138Y_Rv | 5’-AGACGCCCAGGAAGGGATAGTTGCAGAACTGGAAC-3’ |
| Spike_Δ144_Fw | 5’-CCCCTTCCTGGGCGTCTATCACAAGAACAACAA-3’ |
| Spike_Δ144_Rv | 5’-TTGTTGTTCTTGTGATAGACGCCCAGGAAGGGG-3’ |
| Spike_R190S_Fw | 5’-GCAACTTCAAGAACCTGAGCGAGTTCGTGTTCAAG-3’ |
| Spike_R190S_Rv | 5’-CTTGAACACGAACTCGCTCAGGTTCTTGAAGTTGC-3’ |
| Spike_D215G_Fw | 5’-ACCTCGTGCGGGGTCTGCCTCAGGG-3’ |
| Spike_ D215G_Rv | 5’-CCCTGAGGCAGACCCCGCACGAGGT-3’ |
| Spike_A222V_Fw | 5’-TCAGGGCTTCTCTGTTCTGGAACCCCTGG-3’ |
| Spike_A222V_Rv | 5’-CCAGGGGTTCCAGAACAGAGAAGCCCTGA-3’ |
| Spike_K417N_Fw | 5’-CCCTGGACAGACAGGCAATATCGCCGACT-3’ |
| Spike_K417N_Rv | 5’-AGTCGGCGATATTGCCTGTCTGTCCAGGG-3’ |
| Spike_K417T_Fw | 5’-CTGGACAGACAGGCACGATCGCCGACTACAA-3’ |
| Spike_K417T_Rv | 5’-TTGTAGTCGGCGATCGTGCCTGTCTGTCCAG-3’ |
| Spike_N439K_Fw | 5’-GATTGCCTGGAACAGCAAGAACCTGGACTCCAAAG-3’ |
| Spike_N439K_Rv | 5’-CTTTGGAGTCCAGGTTCTTGCTGTTCCAGGCAATC-3’ |
| Spike_L452R_Fw | 5’-GCGGCAACTACAATTACCGGTACCGGCTGTTC-3’ |
| Spike_L452R_Rv | 5’-GAACAGCCGGTACCGGTAATTGTAGTTGCCGC-3’ |
| Spike_Y453F_Fw | 5’-GCGGCAACTACAATTACCTGTTCCGGCTGTTCC-3’ |
| Spike_Y453F_Rv | 5’-GGAACAGCCGGAACAGGTAATTGTAGTTGCCGC-3’ |
| Spike_S477N_Fw | 5’-CTATCAGGCCGGCAACACCCCTTGTAACG-3’ |
| Spike_S477N_Rv | 5’-CGTTACAAGGGGTGTTGCCGGCCTGATAG-3’ |
| Spike_E484K_Fw | 5’-CCCTTGTAACGGCGTGAAAGGCTTCAACTGCTA-3’ |
| Spike_E484K_Rv | 5’-TAGCAGTTGAAGCCTTTCACGCCGTTACAAGGG-3’ |
| Spike_S494P_Fw | 5’-TACTTCCCACTGCAGCCCTACGGCTTTCAGC-3’ |
| Spike_S494P_Rv | 5’-GCTGAAAGCCGTAGGGCTGCAGTGGGAAGTA-3’ |
| Spike_N501Y_Fw | 5’-GGCTTTCAGCCCACATATGGCGTGGGCTATC-3’ |
| Spike_N501Y_Rv | 5’-GATAGCCCACGCCATATGTGGGCTGAAAGCC-3’ |
| Spike_A570D_Fw | 5’-TGGCCGGGATATCGACGATACCACAGACG-3’ |
| Spike_A570D_Rv | 5’-CGTCTGTGGTATCGTCGATATCCCGGCCA-3’ |
| Spike_D614G_Fw | 5’-CAGTGCTGTACCAGGGCGTGAACTGTACCGA-3’ |
| Spike_D614G_Rv | 5’-TCGGTACAGTTCACGCCCTGGTACAGCACTG-3’ |
| Spike_H655Y_Fw | 5’-TGTCTGATCGGAGCCGAGTATGTGAACAATAGCTACGAG-3’ |
| Spike_H655Y_Rv | 5’-CTCGTAGCTATTGTTCACATACTCGGCTCCGATCAGACA-3’ |
| Spike_Q675H_Fw | 5’-CATCTGTGCCAGCTACCATACACAGACAAACAGCC-3’ |
| Spike_Q675H_Rv | 5’-GGCTGTTTGTCTGTGTATGGTAGCTGGCACAGATG-3’ |
| Spike_P681H_Fw | 5’-CACAGACAAACAGCCACAGACGGGCCAGATC-3’ |
| Spike_P681H_Rv | 5’-GATCTGGCCCGTCTGTGGCTGTTTGTCTGTG-3’ |
| Spike_A701V_Fw | 5’-AATGTCTCTGGGCGTCGAGAACAGCGTGG-3’ |
| Spike_A701V_Rv | 5’-CCACGCTGTTCTCGACGCCCAGAGACATT-3’ |
| Spike_T716I_Fw | 5’-CTCTATCGCTATCCCCATCAACTTCACCATCAGCG-3’ |
| Spike_T716I_Rv | 5’-CGCTGATGGTGAAGTTGATGGGGATAGCGATAGAG-3’ |
| Spike_D839Y_Fw | 5’-TCATCAAGCAGTATGGCTATTGTCTGGGCGACATT-3’ |
| Spike_D839Y_Rv | 5’-AATGTCGCCCAGACAATAGCCATACTGCTTGATGA-3’ |
| Spike_D936Y_Fw | 5’-CATCGGCAAGATCCAGTATAGCCTGAGCAGCACAG-3’ |
| Spike_D936Y_Rv | 5’-CTGTGCTGCTCAGGCTATACTGGATCTTGCCGATG-3’ |
| Spike_S982A_Fw | 5’-TGCTGAACGATATCCTGGCCAGACTGGACAAGGTGG-3’ |
| Spike_S982A_Rv | 5’-CCACCTTGTCCAGTCTGGCCAGGATATCGTTCAGCA-3’ |
| Spike_T1027I_Fw | 5’-CCAATCTGGCCGCCATCAAGATGTCTGAGTG-3’ |
| Spike_T1027I_Rv | 5’-CACTCAGACATCTTGATGGCGGCCAGATTGG-3’ |
| Spike_D1118H_Fw | 5’-CCAGATCATCACCACCCACAACACCTTCGTGTC-3’ |
| Spike_D1118H_Rv | 5’-GACACGAAGGTGTTGTGGGTGGTGATGATCTGG-3’ |
| Spike_Q1208H_Fw | 5’-GGGGAAGTACGAGCATTACATCAAGTGGCCC-3’ |
| Spike_Q1208H_Rv | 5’-GGGCCACTTGATGTAATGCTCGTACTTCCCC-3’ |
| GFP_Fw | 5′-TCAGGGATCCACCATGGTGAGCAAGGGCGAG-3′ |
| GFP_Rv | 5′-TCAGCTCGAGTTACTTGTACAGCTCGTCCATGC-3′ |
| ACE2_Fw | 5’-GAGCTCGAGATGTCAAGCTCTTCCTGG-3’ |
| ACE2_Rv | 5’-CGCACGCGTCTAAAAGGAGGTCTGAAC-3’ |
