# Supplementary material for: Signatures in SARS-CoV-2 spike protein conferring escape to neutralizing antibodies
Source: PLoS Pathog. 2021 Aug 5;17(8):e1009772. doi: 10.1371/journal.ppat.1009772 (PMC8341613; doi:10.1371/journal.ppat.1009772)
Supplement: Attachment — Submitted filename: cover letter and answer to reviewers.docx [file ppat.1009772.s018.docx]

Maria João Amorim

Principal Investigator of the

Cell Biology of Viral Infection Lab

Instituto Gulbenkian de Ciência

Rua da Quinta Grande, 6

2780-156 Oeiras, Portugal

**Subject:** Answer to reviewers on the manuscript “*Amino acids 484 and 494 of SARS-CoV-2 spike are hotspots of immune evasion affecting antibody but not ACE2 binding****”.***

Oeiras, 21^st^ July 2021

Dear Michael Diamond, Kasturi Haldar, and esteemed reviewers,

Thank you very much for your comments to our work.

We have revised our manuscript to comply with requests of both reviewers. Reviewer 1 asked for minor alterations that we fully met and to whom we thank for spotting mistakes. We also provided some additional information that was requested regarding the prevalence of circulating viruses in Portugal at the time we collected the sera. Reviewer 2 felt that although being “extremely thorough”, the manuscript “lacked focus and was too long”. Therefore, we have made modifications to transform the manuscript into a more streamlined version.

For the simplified, more focused version, we selected the aspects that both reviewers highlighted as being the novelty of the manuscript. These are three: The first is that both agreed that we presented a detailed analysis of spike variants, harbouring single or combined mutations, and neutralization data for a large panel of variants to explore the importance of single and synergetic mutations in spike. The second is the search for signatures enabling spike to escape neutralizing antibodies by computationally predicting, based on ACE2 and antibody binding information, the amino acids most tolerable for mutations. These are the ones avoiding antibody binding without effecting ACE2 binding and combining mutations amongst amino acids of the different classes is important for enhancing immune evasion. The third is that with this strategy, we identified that mutations in the amino acid sites 484 and 494 are likely sites of immune escape, that combined with mutations that increase binding to ACE2 lead to increased escape of neutralizing antibodies. We validated our findings testing mutations in 484, 494 and 501 with sera from naturally infected and vaccinated people.

As a response to the concerns of reviewer 2, we were asked to lighten the manuscript, by reducing the detail, and we incorporated the suggestions of reviewer 2, as follows:

1) The removal of the geography of the individual variants as these were considered “not particularly useful to the reader”. We agree that the geography is excessive detail, and have removed supplementary FigS1. As a consequence, we reduced the background information of Figure 1, as asked by this reviewer.
2) The presentation of the prevalence of each viral mutation in the form of a table. We think that the prevalence is important in the form of a graph because it allows readers to investigate the dynamic fluctuations and independent emergence of specific single and combined mutations over time. We have, however, simplified the graphs to make them more visually appealing. We hope the reviewer agrees with this simplified version and with the necessity of analysing the dynamic appearance of spike mutations over time.
3) The removal of the numerous structures of the variants, particularly Figures S3-S5 (with individual side chains) as they were considered “unnecessary and the figure legends for these figures contained too much guessing regarding the functions of the individual amino acids”. We removed these Figures, as requested.
4) The reorganization of the neutralization assays data. Reviewer 2 felt that the large number of figures and supplementary figures makes it difficult to follow. We have simplified the supplementary figures to make them easier to follow. However, PLOS pathogens requests all the data to be presented either in a repository or in the manuscript and therefore, for the sake of clarity, we decided to include all the data here, so that everyone may judge how we reached the NT50 values for each engineered spike version. Therefore, we think that the supplementary graphs containing all the neutralization assays go in line with the guidelines of PLOS pathogens. We hope reviewer 2 and editors think this is a valid justification to keep the data here.
5) The change of the title of the paper as the title of the paper emphasizes spike amino acids 484 and 494, which the reviewer felt as having “little to do with the neutralization data presented in the results section”. We changed the title to *Signatures in SARS-CoV-2 spike protein conferring escape to neutralizing antibodies****.***

With these changes we hope that reviewers find the results cohesively explained in a tied, streamline story.

All requested files have been included in the submission system.

Best regards,

**Authors response to reviewers**

- We thank the reviewers for taking the time to read our manuscript and for providing valuable suggestions. We altered the manuscript to comply with both reviewers and hopefully reviewer 2 will find that the manuscript contains the same thorough and detailed information in a more streamlined version with a matching title and content. We split the comments per reviewer and answered each reviewer’s question separately. Answers are in blue in sentences that start with an arrow (🡪), for making it easy to track.

**Reviewer #1:**

- We thank reviewer 1 for the nice comments about our work. We complied with the suggestions and answered the questions below each comment.

**Part I - Summary**
Please use this section to discuss strengths/weaknesses of study, novelty/significance, general execution and scholarship.

Reviewer #1: SARS-CoV-2 variants which escape antibody neutralization are of significant concern as previous studies have demonstrated that the current vaccines induce humoral immunity with less neutralization capacity against these variants. Here, the authors use a lentiviral spike-pseudotyped reporter viral particle to characterize polyclonal neutralization against the most prevalent variants of concern. Additionally, the authors computationally predict, based off ACE2 and antibody binding, the amino acids most tolerable for mutations that can avoid antibody binding without effecting ACE2 binding. With this strategy, the authors have identified amino acid position 484 and 494 as likely to emerge in variant strains. These two amino acid sites have previously been characterized as hotspots for antibody escape mutants. The authors combine these specific amino acid mutations to quantify synergistic effect on neutralizing antibody responses.

Overall, this study recapitulates many of the previous results demonstrating mutational hotspots that give rise to viral mutants able to escape antibody neutralization. The unique aspect of this study is combining multiple mutations into a single reporter viral constructs to characterize the synergistic effect of specific amino acid mutations on serum neutralization capacity. Through these efforts, the authors identify that mutations in the amino acid sites 484 and 494 as the most likely sites for synergistic mutations that escape antibody neutralization. This manuscript is very clearly written and the results support the conclusions. Below is specific, minor critique.

- Thank you. We provide answers to minor concerns below.

**Part II – Major Issues: Key Experiments Required for Acceptance**
Please use this section to detail the key new experiments or modifications of existing experiments that should be absolutely required to validate study conclusions.

Generally, there should be no more than 3 such required experiments or major modifications for a "Major Revision" recommendation. If more than 3 experiments are necessary to validate the study conclusions, then you are encouraged to recommend "Reject".

Reviewer #1: (No Response)

**Part III – Minor Issues: Editorial and Data Presentation Modifications**
Please use this section for editorial suggestions as well as relatively minor modifications of existing data that would enhance clarity.

Reviewer #1: Human Sera: Do the authors know the strain of the virus infecting patients from which sera was isolated? If the strain is not conclusively known, can the authors speculate on the strain based off the dominant circulating strains in Portugal at the time of blood draw? The infecting strain will likely effect the specificity and target of the humoral immune response.

- We do not have information on the virus infecting patients from which sera was isolated. We do know, however, that sera was collected between Spring/Summer of 2020 and we plotted the frequency of the variants found in Portugal during this period. During this period, the variant B.1.1 encoding WT spike was prevalent.

Prevalence of SARS-CoV-2 lineages in Portugal

Frequency (%)

|  | B.1.1 | B.1 | B.1.91 | B.40 | B | B.1.1.421 | B.1.1.401 | B.1.1.410 | B.1.1.269 | B.1.1.198 |
| --- | --- | --- | --- | --- | --- | --- | --- | --- | --- | --- |
| March | 41.09 | 22.17 | 19.38 | 3.49 | 0 | 0 | 0 | 0 | 0 | 0 |
| April | 40.08 | 30.58 | 14.88 | 0 | 4.55 | 0 | 0 | 0 | 0 | 0 |
| May | 59.14 | 17.2 | 0 | 0 | 0 | 5.38 | 0 | 4.3 | 0 | 0 |
| June | 40 | 14.29 | 0 | 0 | 0 | 14.29 | 13.33 | 5.71 | 6.67 | 5.71 |

**Graph 1** – Prevalence of variants circulating in Portugal from 1^st^ of March to 30^th^ June 2020 with incidence above 2.5%.

- In the manuscript, we added the sentence highlighted in yellow to refer to this in the materials and methods section: Human convalescent sera and post-vaccination plasma/serum: Venous blood was collected by standard phlebotomy from health care providers who contracted COVID-19 during spring/summer 2020, as tested by RT-PCR on nasopharyngeal swabs. During this period, the variant B.1.1 was prevalent in Portugal, with encodes the WT spike protein.

Line 63-64. The cascade of spike processing is a little off here. S1/S2 is cleaved by the host protease furin during viral egress. The S2’ is cleaved after viral attachment by host protease TMPRSS2 on the cell surface. PMID: 32362314

- We corrected this.

Line 321-327: The spike-pseudotyped lentiviral GFP reporter particle is not novel and has been developed by others. (Summarized in PMID: 32384820)

- We aimed at highlighting that the method of analysis was suitable, not the spike-pseudotyped lentiviral GFP reporter itself as novel. In fact, we even cite a GFP based spike-pseudotyped particle in the following manscript, that is very similar to ours.

**Reference:** Schmidt F, Weisblum Y, Muecksch F, Hoffmann H-H, Michailidis E, Lorenzi JCC, et al. Measuring SARS-CoV-2 neutralizing antibody activity using pseudotyped and chimeric viruses. Journal of Experimental Medicine. 2020;217(11).

To make this clear we have corrected the text as follows (differences highlighted in yellow): Our method of reading fluorescent signals in 96 wells plates using the GloMax Explorer System is suitable to assess the neutralization activity of sera/plasma from individuals infected naturally or vaccinated, and to screen for antiviral drugs that block viral entry, such as therapeutic antibodies, in biosafety level 2 settings, which greatly facilitates the procedure and broadens its usage.

Line 114: skype -> spike

- Corrected.

Line 140: SA (South Africa)

- This part has been removed from the manuscript in the new version to accommodate the request of reviewer 2 of simplifying the story.

Line 287: covalence -> convalescent

- Corrected.

**Reviewer #2:**

**Part I - Summary**
Please use this section to discuss strengths/weaknesses of study, novelty/significance, general execution and scholarship.

- We thank this reviewer for the constructive comments. We accommodated the concerns as much as possible, aiming at providing less detail but maintaining the thoroughness of the manuscript, whilst providing a coherent streamlined story. For the focused version, we selected three aspects. The first is the detailed analysis of spike variants, harbouring single or combined mutations, and neutralization data for a large panel of variants to explore the importance of synergetic mutations. The second is the search for signatures enabling spike to escape neutralizing antibodies by computationally predicting, based on ACE2 and antibody binding information, the amino acids most tolerable for mutations. These are the ones avoiding antibody binding without effecting ACE2 binding and that combining the mutations of the two classes augments the capacity of escaping neutralization. The third is that with this strategy, we identified that mutations in the amino acid sites 484 and 494 as likely sites for synergistic mutations that escape antibody neutralization and we tested them with sera from naturally infected and vaccinated people.

Reviewer #2: The study from Alenquer et al. investigates the interplay between SARS-COV-2 variants and neutralizing antibodies/ spike protein. The manuscript presents three main topics: 1) analysis of SARS-COV-2 variants throughout the world, resulting in identification of a panel of emerging variants of concern with mutations in the spike protein. 2) Creation of a panel of spike-pseudotyped viruses expressing wild type and mutant spike proteins, which are then used in neutralizing antibody assays with convalescent and vaccine-immune sera to assess sensitivity to neutralization. 3) use of existing virus-spike and virus-antibody structures to determine spike protein residues frequently engaged with antibodies and/or ACE-2 receptor. One conclusion of the study is the identification of spike mutants E484K and S494 as positions that engage with neutralizing antibodies, but not ACE-2, and are thus prone to immune escape.

**Part II – Major Issues: Key Experiments Required for Acceptance**
Please use this section to detail the key new experiments or modifications of existing experiments that should be absolutely required to validate study conclusions.

Reviewer #2: Overall, the paper is extremely thorough, but lacks focus and is too long. Many of the variants studied (B.1.1.7, B.1.351) were already known, and the descriptions of the variants in supplementary Table S1 indicate that neutralization sensitivity and/or ACE-2 binding has been studied already.

- We acknowledge that many mutations or even complete variants in spike were already presented in individual studies. However, the novelty of this paper is presented in 2 ways. First, by studying a rich set of mutations in a single study, we can make comparisons amongst mutations, which has not done thoroughly. In addition, we study sets of mutations not investigated before to understand how combining mutations changes their recognition by neutralizing antibodies. Second, we mimicked the entire set of mutations in the protein spike of variants of concern. This has not been done before and is different from using the “real virus” because these variants also have mutations in other ORFs able to, for example, change the speed of replication.
- Regarding the Table S1. although, we highlight what is known for each individual mutation or combination of mutations presented in our engineered mutant, this does not mean that the mutant spike under study has been analysed before. For example, we engineered the triple mutant Δ69H-70V/N439K. A lot is known about the double deletion and about the N439K mutation, but from separate studies and no information was known from the triple mutant. This is just one example, and other sets of mutations in spike are unique in this study including:
- Δ69H-70V/Δ144/N501Y/A570D/P681H/T716I/S982A/D1118H;
- D80A/D215G/K417/E484K/N501Y/A701V;
- L18F/T20N_P26S/D138Y/R190S/K417T/E484K/N501Y/H655Y/T1027I;
- Δ69H-70V/N501Y/ P681H; E484K/S494P/N501Y.

The sequence analysis presented is very detailed, and perhaps beyond the scope of the study. The first paragraph of the results contains too much background information, and the geography/prevalence of the individual variants is not particularly useful to the reader and could be presented more succinctly in table form.

- We simplified the sequence analysis in the text in several ways. First, we agree that the geography is excessive detail, and have removed supplementary FigS1. As a consequence, we reduced the background information, as asked. As for the prevalence of each viral mutation, we think that the prevalence is important in the form of a graph because it allows readers to investigate the dynamic fluctuations and independent emergence of specific single and combined mutations over time. We have simplified the graphs to make them more visually appealing. We hope the reviewer agrees with this simplified version and with the necessity of analysing the dynamic appearance of spike mutations over time.

Furthermore, the numerous structures of the variants, particularly Figures S3-S5 (with individual side chains) are unnecessary and the figure legends for these figures contain too much guessing regarding the functions of the individual amino acids.

- We removed these Figures, as requested.

The neutralization assay data is rigorous, but the large number of figures and supplementary figures is difficult to follow.

- We have simplified the supplementary figures to make them easier to follow. However, PLOS pathogens requests all the data to be presented either in a repository or in the manuscript and therefore, for the sake of clarity, we decided to include all the data here, so that everyone may judge how we reached the NT50 values for each engineered spike version. Therefore, we think that the supplementary graphs containing all the neutralization assays go in line with the guidelines of PLOS pathogens. We hope reviewer 2 and editors think this is a valid justification to keep the data here.

The major conclusions of the paper are based on published structural data (available spike-mAb & spike-ACE-2 complexes). While the title of the paper emphasizes spike amino acids 484 and 494, this has little to do with the neutralization data presented in the results section. Overall, the authors have presented a detailed analysis of viral variants, neutralization data for a large panel of spike variants, and an analysis of the current structural data regarding spike residues targeted by neutralizing antibodies versus ACE-2. However, these results are not tied together in a cohesive story.

- We changed the title to ***Signatures in SARS-CoV-2 spike protein conferring escape to neutralizing antibodies.***
